# Demographic, Behavioral, and Psychosocial Correlates of Using the Website Component of a Worksite Physical Activity and Healthy Nutrition Promotion Program: A Longitudinal Study

**DOI:** 10.2196/jmir.1402

**Published:** 2010-09-30

**Authors:** Suzan JW Robroek, Wendy Brouwer, Dennis Lindeboom, Anke Oenema, Alex Burdorf

**Affiliations:** ^2^Lifeguard IncUtrechtNetherlands; ^1^Department of Public HealthErasmus MC, University Medical CenterRotterdamNetherlands

**Keywords:** Internet, physical activity, nutrition, behavior change, selective retention, workplace

## Abstract

**Background:**

Internet-delivered behavior change programs have the potential to reach a large population. However, low participation levels and high levels of attrition are often observed. The worksite could be a setting suitable for reaching and retaining large numbers of people, but little is known about reach and use of Internet-delivered health promotion programs in the worksite setting.

**Objective:**

This study aimed (1) to gain more insight in the use of the website component of a worksite behavior change intervention and (2) to identify demographic, behavioral, and psychosocial factors associated with website use.

**Methods:**

The study was an observational study among participants from 5 workplaces in a cluster randomized controlled trial. At baseline, all participants visited a study website to fill out the baseline questionnaire. Then a physical health check was done followed by face-to-face advice. After this contact, all participants received an email to promote visiting the website to view their health check results and the personal advice based on the baseline questionnaire. In the subsequent period, only participants in the intervention group received monthly email messages to promote website visits and were offered additional Web-based tools (self-monitors and a food frequency questionnaire [FFQ] assessing saturated fat intake) to support their behavior change. Website use was monitored by website statistics registering website access. Complete data were available for 726 employees. Logistic regression analyses were conducted to identify characteristics of employees who visited and used the website.

**Results:**

In total, 43% of the participants visited the website after the email to promote website visits. Participants who were insufficiently physically active were less likely to visit the website (odds ratio [OR] 0.63, 95% confidence interval [CI] 0.45-0.88), whereas individuals with an elevated total cholesterol level visited the website more often (OR 1.44, 95% CI 1.05-1.98). The monthly emails in the intervention group resulted in higher website use during a 3-month period (18% versus 5% in the reference group, OR 3.96, 95% CI 2.30-6.82). Participants with a positive attitude toward increasing physical activity were less likely to visit the website (OR 0.54, 95% CI 0.31-0.93) or to use the self-monitor and FFQ (OR 0.50, 95% CI 0.25-0.99). Female workers visited the website more often to monitor their behavior and to receive advice on fat intake (OR 2.36, 95% CI 1.14-4.90).

**Conclusions:**

Almost half of the participants used the website component of a worksite behavior change program. Monthly emails were a prompt to visit the website, but website use remained low. More women than men used the website to obtain personalized advice for behavior change. No consistently higher participation was found among those with healthier behaviors. This health promotion program did not provide an indication that healthier subjects are more susceptible to health promotion.

**Trial Registration:**

ISRCTN52854353; http://www.controlled-trials.com/ISRCTN52854353 (Archived by WebCite at http://www.webcitation.org/5smxIncB1)

## Introduction

There are indications that Internet-delivered interventions may be effective in improving physical activity, healthy nutrition, and weight reduction [1-5]. Internet-delivered programs have the potential to reach a large population at relatively low costs. However, low participation levels and high levels of attrition are often observed in those programs [5-8]. These rates are of concern since studies with a higher utilization tend to have better behavior change outcomes [5]. The RE-AIM framework stresses the importance of evaluating the reach and representativeness of program participants [9], and Eysenbach [6] and Danaher et al [10] have emphasized the need to address process measures in addition to the effectiveness of Internet-delivered programs. The worksite has been identified as a promising setting to reach large numbers of people in a natural social network, which may increase participation [11,12]. However, the reach and use of Internet-delivered programs in the worksite setting are largely unknown.

In contrast with the high levels of attrition in the general population, Ware et al [13] studied an intervention consisting of an Internet-delivered program at the worksite with an initial face-to-face contact and found a repeated participation over a 12-week period of 69%. Several studies on Internet-delivered behavior change programs suggested that women, people who are more highly educated, and people with positive health behaviors participate more often in Internet-delivered health promotion programs compared with the general population [8,14-16]. However, there are also studies indicating that Internet-delivered programs have attracted individuals who would benefit most from them, that is, participants who are overweight [8,13,16]. It has also been suggested that the provision of regular new content and the possibility to monitor progress toward behavior change could be important factors in encouraging website use [17,18]. Furthermore, a recent review reported several studies with enhanced effectiveness after frequent email prompts [19].

It has been indicated that participants may not be ready to rely solely on Internet-delivered programs [5]. The worksite setting, in which it is feasible to combine face-to-face contact and regular emails, may, therefore, be a good setting for the implementation of interventions. Therefore, we expect that providing an Internet-delivered lifestyle program in the workplace setting with an initial face-to-face contact, a behavior change monitor functionality, and monthly email messages will enhance program use.

More insight into these specific program characteristics could provide information on ways to attract visitors to an Internet-delivered health promotion intervention and to keep them using the program. The aim of the present study is to gain more insight into the use of a website component of a worksite intervention, in order to be able to identify factors related to website use and intervention components that may enhance use. Therefore, the present study investigates the demographic, behavioral, psychosocial, and health-related factors in relation to program use in an Internet-delivered program with a face-to-face contact at the worksite.

## Methods

### Design, Participants, and Recruitment

An observational study was conducted from March 26, 2008, until February 9, 2009. Participants were employees from 5 different workplaces: 2 companies engaged in commercial services, 2 in health care, and 1 executive branch of government. The participants had enrolled in a 2-year cluster randomized controlled trial in which the departments (64) within these 5 workplaces were the units of randomization. An extensive description of this larger worksite lifestyle promotion program primarily aimed at physical activity and nutrition is described elsewhere [20]. The study was announced through email, the company’s intranet and/or a company magazine. In the 2 commercial services companies, all employees directly received an email from a health management organization that had implemented the intervention in which employees were invited to visit the study website. For the other workplaces, interested employees could express their interest in participating in the study through email. These 3 workplaces restricted the maximum number of participants in such a way that the first 200 (2 workplaces) or 300 (1 workplace) interested employees were allowed to participate. Participants enrolled in the study when they visited the website and completed the baseline questionnaire. Participation levels varied from 3% to 61% of all workers per workplace, with a median participation level of 10%. The number of participants per workplace ranged from 33 to 270 (median 175), and workplace sizes varied from 70 to more than 5000 employees (median 1706). Complete data on individual characteristics, behaviors, and health were available for 726 employees. The Medical Ethics Committee of Erasmus MC, University Medical Center in Rotterdam, the Netherlands, approved the study and all participants gave written informed consent.

**Figure 1:**
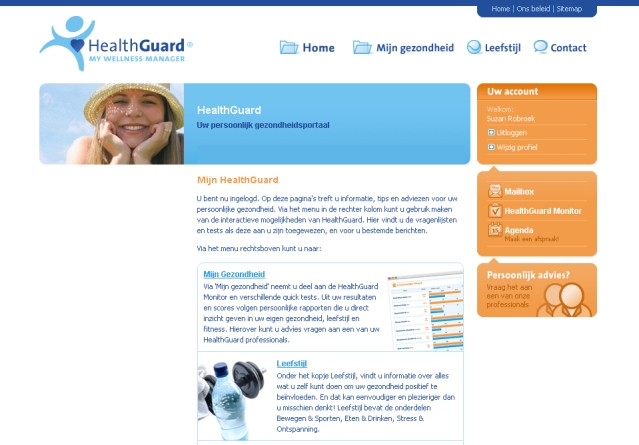
Screenshot of the website

### Procedure

**Figure 2:**
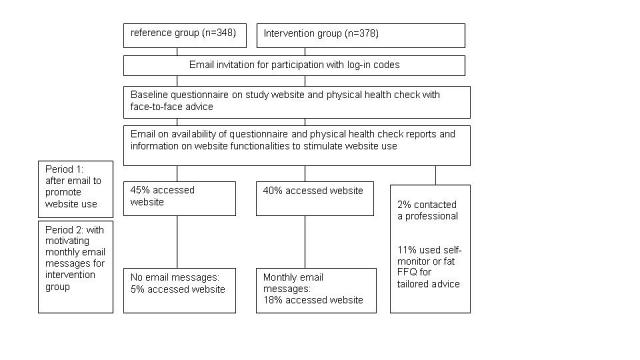
Study design with the two distinct periods for website use

All participants visited the study website by using an individualized username and password to fill out the baseline questionnaire and to make an appointment for a physical health check (Figure 1). The health check took place at the workplace and consisted of measurement of height, weight, waist circumference, total cholesterol level, blood pressure, and a bicycle test to estimate maximum oxygen uptake. Immediately after the health check, all participants received an overview of their test results in print. These results were discussed with the participants, and each participant received advice on how to improve or maintain their lifestyle in a face-to-face contact. Participants who were prehypertensive or who had an elevated cholesterol level were advised to visit their general practitioner or the occupational physician. The physical health check took one hour, and workers were allowed to participate during their regular work hours. The test reports were also provided on the study website together with personal advice based on participants’ answers on the baseline questionnaire. After all participants in one workplace had completed the health checks, all participants were invited through an email message to visit the website to view their health check results and the personal advice based on the baseline questionnaire (see Figure 2, period 1). The personal advice provided on the website corresponded with the advice in the face-to-face contact and was provided in a structured and reproducible way.

#### Reference Group

Participants in the reference group had access to their physical health results and reports based on the online questionnaire. These reports consisted of their personal physical activity level and fruit and vegetable intake level and information on the recommended levels. The website provided general lifestyle and health information.

#### Intervention Group

Participants in the intervention group had access to several additional website functionalities compared with participants in the reference group. Participants in the intervention group received more extensive computer-tailored advice on their self-reported physical activity and nutrition behavior on the questionnaire. The electronically generated advice included personal and action feedback taking into account perceived barriers for participants not meeting the guidelines [20,21]. Perceived barriers were assessed by asking for the most important barrier to engaging in the specific lifestyle behavior.

In addition, participants had the opportunity to use the following intervention strategies: (1) online self-monitoring of fruit and vegetable intake, physical activity, and weight to monitor progress toward behavior change and obtain tracking charts; (2) a food frequency questionnaire (FFQ) assessing saturated fat intake for tailored advice (after third email message) [22]; and (3) the ability to ask questions of several professionals.

Finally, to stimulate sustained website use, participants in the intervention group received motivating monthly email messages focusing on physical activity and nutrition. Participants received their first motivating email message 1 month after they received an email to visit the website to view their health check results and the advice based on the baseline questionnaire. With the motivating email messages, the second important period of the website component started (see Figure 2, period 2). Period 2 covered 3 monthly email messages focusing on physical activity and nutrition (duration of 12 weeks). The first monthly email message was tailored to the individual, and if new information was available through the self-monitors, the subsequent email was personalized again. If no new information from the participant was available, the emails contained more general information. The third message announced the opportunity to fill out the fat FFQ for tailored advice. In all monthly email messages, participants were encouraged to fill out the self-monitors and to ask their questions. The monthly email messages were written by a researcher (author SR).

#### Website Use

Participants had to log in to the website using their personal login details to access their individual reports as well as to read general information on health and lifestyle. All website visits were registered, and for both period 1 and period 2, a variable for website visit (yes/no) was calculated for all the participants. Website use in period 1 was determined as at least 1 website visit within the month after the email was sent to promote website use. Website use in period 2 was determined as at least 1 website visit within 3 months after the first motivating monthly email message to the intervention group. For participants in the intervention group, self-monitor use and fat FFQ use were defined as using these features at least once in period 1 or period 2.

#### Demographic Characteristics

In the baseline questionnaire, participants were asked about age, sex, education, marital status, and ethnicity. Educational level was assessed as the highest level of education completed and was categorized into low (primary school, lower and intermediate secondary schooling, or lower vocational training), intermediate (higher secondary schooling or intermediate vocational schooling) and high (higher vocational schooling or university). We applied the standard definition of ethnicity of Statistics Netherlands and considered a person to be nonDutch if at least one parent was born abroad [23].

#### Lifestyle Behavior and Health Indicators

Physical activity level was measured in the baseline questionnaire using the self-administered short version of the International Physical Activity Questionnaire (IPAQ) [24], which assessed vigorous and moderate intensity physical activity. The average time spent on physical activity per day was calculated. For all behaviors we calculated a dichotomous variable for compliance or noncompliance with recommendations. For physical activity level, we used a cutoff point of 30 minutes or more per day. We did not include walking in this calculation since walking at a casual pace is regarded a light-intensity activity [25].

For fruit and vegetable intake, 400 grams of fruit and vegetable intake as measured with a self-administered 9-item validated Dutch Food Frequency Questionnaire was used as cutoff point [26]. Smoking was defined as current smoking status and excessive alcohol use as drinking at least 6 glasses on the same occasion at least once a week. The Short Form-12 (SF-12) questionnaire was used to measure self-reported general, physical, and mental health [27]. General health was dichotomized into “poor or moderate” and “good to excellent.” Physical and mental health were categorized as poor if the summed scores were in the lowest quartile (lower than 48.74 and 46.56, respectively).

##### Physical Health Check

In the physical health check, height and weight were measured to calculate body mass index (BMI) and to categorize individuals as normal weight (BMI < 25 kg/m^2^) or overweight (BMI ≥ 25 kg/m^2^). Total blood cholesterol was measured in nonfasting blood through a finger prick (Accutrend GC, Roche Company, Mannheim, Germany), and blood pressure with a fully automated sphygmomanometer (Omron M4-I, Omron HealthCare Europe BV, Hoofddorp, the Netherlands). A total cholesterol level above 5.0 mmol/l and a systolic or diastolic blood pressure above respectively 140 mmHg and 90 mmHg were considered elevated. A submaximal exercise test on a bicycle ergometer was conducted to predict maximal oxygen uptake according to the American College of Sports Medicine’s (ACSM) protocol using their sex- and age-dependent cutoff points [28].

##### Social Cognitive Variables

For physical activity and for fruit and vegetable intake, attitude, social support, self-efficacy, and intention to change were measured in the baseline questionnaire. Intention, self-efficacy, and attitude were measured on a 5-point Likert scale ranging from “certainly not” to “certainly.” All variables were dichotomized. Intention was measured by asking if the participant intended to change the behavior in the next month [29]. A high intention was defined as probably or certainly intending to change the behavior. Self-efficacy was assessed by asking if the participant was confident about engaging in the healthy behaviors in the next month [29]. High self-efficacy was defined as probably or certainly confident about changing the behavior. To measure attitude, individuals were asked if they thought improving the behavior would take a lot of effort [30]. Those participants who answered “probably not” or “certainly not” were considered as having a positive attitude. Finally, social support was measured by asking if family and friends support them in changing the specific behaviors. This was measured on a 4-point Likert scale ranging from “seldom or never” to “a lot” [29]. High social support was defined as perceiving “pretty much” support or “a lot” of support.

#### Statistical Analyses

Descriptive statistics were used to present the baseline characteristics of the study population. The associations of demographic characteristics, behaviors, social cognitive variables, and health indicators with website use were investigated with logistic regression analysis. Separate analyses were conducted for website use in period 1 among the total study population and website use in period 2 among the intervention group. First, univariate logistic regression models were carried out to determine the single effects of the possible determinants. All variables with a *P* value less than .20 in the univariate models were considered for inclusion in the multivariate analysis. A backward regression method was used to determine the multivariate model. In the analyses, age and sex were included by default in each multivariate model. Variables with a *P* value of .05 or less were retained in the multivariate model. The results are presented as the odds ratios (OR) and corresponding 95% confidence intervals (95%CI), with odds ratios below and above 1 representing, respectively, lower and higher website use. All analyses were carried out with SPSS version 15.0 (SPSS Inc, Chicago, IL, USA).

## Results

### Study Population

In total, 726 employees participated in this study. The baseline characteristics of the study population are presented in Table 1. More than half of the participants (403, 56%) were female workers. The mean age of the study population was 42 years, ranging from 20 to 63 years, and 47% (341) had had higher education. Almost a third of the participants (223, 31%) were not physically active at a moderate intensity for at least 30 minutes per day, and 45% (323) had insufficient fruit and vegetable intake. Complying with the moderate intensity physical activity guideline was associated with sufficient fruit and vegetable intake (not in table). More than half of the participants who did not met the physical activity guideline for moderate intense physical activity had the intention to increase physical activity (122/223, 55%), compared with 45% (225/503) of the participants who did comply with the guideline. For fruit and vegetable intake, 22% (71/323) of the participants who did not meet the recommendation and 13% (52/403) of the participants who did, intended to increase fruit and vegetable intake. Participants complying with the guidelines were more likely to have a positive attitude. No association was found between self-efficacy and complying to the recommended levels for physical activity and fruit and vegetable intake (not in table).

### Website Visit

After the first email message, 43% of all the participants visited the website component of the program; 45% (157/348) of the participants in the reference group and 40% (152/378) in the intervention group (odds ratio [OR] 0.82, 95% confidence interval [CI] 0.61-1.10). In the following three months in which the intervention group received a monthly email message, 18% (67/378) of the participants in the intervention group visited the website again compared with 5% (18/348) in the reference group (OR 3.96, 95% CI 2.30-6.82).

**Table 1:** Baseline characteristics of the total study population and the intervention group in a longitudinal study among 726 employees

			Total Study Population (n = 726)		Intervention Group (n = 378)		Reference Group (n = 348)	
			n	%	n	%	n	%
**Demographics**								
	Female gender		403	56%	209	55%	194	56%
	**Age (years)**							
		<30	100	14%	56	15%	44	13%
		30-39	203	28%	94	25%	109	31%
		40-49	228	31%	112	30%	116	33%
		50+	194	27%	115	31%	79	23%
	**Education level**							
		Low	131	18%	60	16%	71	20%
		Intermediate	253	35%	131	35%	122	35%
		High	341	47%	186	49%	155	45%
	Dutch ethnicity		615	85%	319	85%	296	85%
	Married/cohabiting		547	75%	285	76%	262	75%
	**Behavior**							
		Insufficient moderate physical activity	223	31%	115	31%	108	31%
		Insufficient vigorous physical activity	502	69%	258	68%	244	70%
		Insufficient fruit or vegetable intake	323	45%	159	42%	164	47%
		Smoking	117	16%	60	16%	57	17%
		Excessive alcohol	27	4%	13	3%	14	4%
**Social cognitive variables**								
	**Physical activity**							
		Positive attitude	355	49%	197	52%	158	45%
		High social support	112	15%	55	15%	57	16%
		High self-efficacy	562	77%	288	76%	274	79%
		Intention to increase physical activity	348	48%	167	44%	181	52%
	**Fruit and vegetable intake**							
		Positive attitude	510	70%	265	70%	245	71%
		High social support	91	13%	46	12%	45	13%
		High self-efficacy	599	83%	319	84%	280	81%
		Intention to increase intake	124	17%	68	18%	56	16%
	**Health indicators**							
		Overweight/obese	293	40%	152	40%	141	41%
		Poor/moderate general health	39	5%	17	5%	22	6%
		Lowest quartile mental health	181	25%	97	26%	84	24%
		Lowest quartile physical health	181	25%	90	24%	91	26%
		Elevated blood pressure	217	30%	113	30%	104	30%
		Elevated total cholesterol level	301	42%	161	43%	140	41%
		Poor predicted maximum oxygen uptake	90	13%	43	12%	47	15%

### Correlates of Website Visit

As shown in the univariate analysis in Table 2, older employees (OR 1.89, 95% CI 1.15-3.13), those with a positive attitude toward increasing physical activity level (OR 1.36, 95% CI 1.01-1.83), and those with an elevated cholesterol level (OR 1.51, 95% CI 1.12-2.04) were more likely to visit the website after the first email message, and participants with insufficient moderate-intensity physical activity (OR 0.66, 95% CI 0.47-0.91) were less likely to do so. In the multivariate analysis, sufficient moderate physical activity (OR 0.64, 95% CI 0.46-0.90 for insufficient physical activity) and an elevated cholesterol level (OR 1.44, 95% CI 1.05-1.98) remained significantly associated with website visit in period 1. Attitude to increase physical activity did not remain statistically significant in the multivariate analysis (OR 1.34, 95% CI 0.98-1.82). Table 3 shows that among the participants in the intervention group, those with a positive attitude toward increasing their level of physical activity (OR 0.57, 95% CI 0.33-0.97) and fruit and vegetable intake (OR 0.55, 95% CI 0.32-0.96) were less likely to visit the website in the period with monthly email messages. In the multivariate analysis, only attitude toward increasing physical activity level (OR 0.54, 95% CI 0.31-0.93) remained statistically significant.

**Table 2:** Univariate and multivariate odds ratios and 95% confidence intervals of individual characteristics, behaviors, social cognitive variables, and health indicators for visiting the website in the first period after the health check (n = 726)

			Univariate Analysis		Multivariate Analysis	
			OR	95% CI	OR	95% CI
**Demographics**						
	Female gender		0.93	0.69-1.25	1.00	0.74-1.36
	**Age (years)**					
		<30	1.00		1.00	
		30-39	1.35	0.82-2.23	1.35	0.81-2.24
		40-49	1.35	0.83-2.21	1.18	0.72-1.96
		50+	1.89^b^	1.15-3.13	1.65	0.97-2.79
	**Education level**					
		Low	0.92	0.62-1.39		
		Intermediate	0.76^a^	0.55-1.06	
		High	1.00			
	Dutch ethnicity		0.96	0.64-1.45		
	Married/cohabiting		1.34^a^	0.94-1.89		
	**Behavior**					
		Insufficient moderate physical activity	0.66^b^	0.47-0.91	0.64^b^	0.46-0.90
		Insufficient vigorous physical activity	1.01	0.73-1.39		
		Insufficient fruit or vegetable intake	1.01	0.75-1.36		
		Smoking	0.71^a^	0.47-1.07		
		Excessive alcohol consumption	0.83	0.37-1.85		
**Social cognitive variables**						
	**Physical activity**					
		Positive attitude	1.36^b^	1.01-1.83		
		High social support	0.84	0.55-1.27		
		High self-efficacy	1.00	0.71-1.43		
		Intention to increase physical activity	1.11	0.83-1.49		
	**Fruit and vegetable intake**					
		Positive attitude	1.22	0.88-1.69		
		High social support	0.97	0.62-1.52		
		High self-efficacy	0.89	0.60-1.31		
		Intention to increase intake	0.70^a^	0.47-1.05		

	**Health indicators**					
		Overweight/obese	0.96	0.71-1.30		
		Poor/moderate general health	1.29	0.68-2.46		
		Lowest quartile mental health	1.18	0.84-1.66		
		Lowest quartile physical health	0.97	0.69-0.37		
		Elevated blood pressure	0.82	0.59-1.13		
		Elevated total cholesterol level	1.51^b^	1.12-2.04	1.44^b^	1.05-1.98
		Poor predicted maximum oxygen uptake	0.83	0.53-1.31		

^a^ *P* < .20, considered for inclusion in the multivariate logistic regression analysis

^b^ *P* < .05

**Table 3:** Characteristics of the intervention group and univariate and multivariate odds ratios and 95% confidence intervals of individual characteristics, behaviors, social cognitive variables, and health indicators for visiting the website in the second period in the intervention group (n = 378)

			Univariate Analysis		Multivariate Analysis	
			OR	95% CI	OR	95% CI
**Demographics**						
	Female gender		1.32	0.77-2.27	1.35	0.78-2.33
	**Age (years)**					
		<30	1.00		1.00	
		30-39	0.97	0.39-2.39	1.02	0.41-2.54
		40-49	1.26	0.54-2.97	1.47	0.62-3.52
		50+	1.14	0.49-2.69	1.37	0.57-3.28
	**Education level**					
		Low	0.57	0.24-1.36		
		Intermediate	1.04	0.59-1.84		
		High	1.00			
	Dutch ethnicity		1.05	0.50-2.20		
	Married/cohabiting		1.01	0.54-1.87		
	**Behavior**					
		Insufficient moderate physical activity	1.06	0.60-1.87		
		Insufficient vigorous physical activity	0.86	0.49-1.51		
		Insufficient fruit or vegetable intake	1.45^a^	0.85-2.46		
		Smoking	0.46^a^	0.19-1.13		
		Excessive alcohol consumption	0.41	0.05-3.24		
**Social cognitive variables**						
	**Physical activity**					
		Positive attitude	0.57^b^	0.33-0.97	0.54^b^	0.31-0.93
		High social support	0.80	0.36-1.78		
		High self-efficacy	0.83	0.45-1.51		
		Intention to increase physical activity	1.11	0.65-1.89		
	**Fruit and vegetable intake**					
		Positive attitude	0.55^b^	0.32-0.96		
		High social support	0.42^a^	0.14-1.20		
		High self-efficacy	1.07	0.51-2.25		
		Intention to increase intake	0.89	0.44-1.80		

	**Health indicators**					
		Overweight/obese	1.27	0.75-2.17		
		Poor/moderate general health	0.99	0.28-3.54		
		Lowest quartile mental health	0.65^a^	0.34-1.24		
		Lowest quartile physical health	1.01	0.55-1.89		
		Elevated blood pressure	0.75	0.41-1.38		
		Elevated total cholesterol level	0.89	0.52-1.52		
		Poor predicted maximum oxygen uptake	0.56	0.21-1.47		

^a^ *P* < .20, considered for inclusion in the multivariate logistic regression analysis

^b^ *P* < .05

### Use of Interactive Website Elements in the Intervention Condition

Of the website visitors in the intervention group, 11% (41/378) used the self-monitors or the FFQ, and 2% (8/378) contacted a professional via the website (Figure 2). Table 4 shows that female workers were more likely to use the self-monitor or fat FFQ compared with male workers (OR 2.36, 95% CI 1.14-4.90). As for website use in period 2, those workers with a positive attitude toward increasing their physical activity level were less likely to visit the website to use the specific website functionalities (OR 0.50, 95% CI 0.25-0.99).

**Table 4:** Univariate and multivariate odds ratios and 95% confidence intervals of individual characteristics, behaviors, social cognitive variables, and health indicators for self-monitor and fat FFQ use in the intervention group (n=378)

			Univariate Analysis		Multivariate Analysis	
			OR	95% CI	OR	95% CI	
**Demographics**						
	Female gender		2.41^b^	1.17-4.96	2.36^b^	1.14-4.90
	**Age (years)**					
		<30	1.00		1.00	
		30-39	0.93	0.34-2.55	0.99	0.36-2.77
		40-49	0.92	0.35-2.45	1.09	0.40-2.98
		50+	0.67	0.24-1.86	0.85	0.30-2.43
	**Education level**					
		Low	0.87	0.34-2.28		
		Intermediate	0.94	0.46-1.93		
		High	1.00			
	Dutch ethnicity		1.77	0.61-5.17		
	Married/cohabiting		1.00	0.47-2.13		
	**Behavior**					
		Insufficient moderate physical activity	1.21	0.61-2.40		
		Insufficient vigorous physical activity	1.00	0.50-2.01		
		Insufficient fruit or vegetable intake	1.69^a^	0.88-3.24		
		Smoking	0.54	0.19-1.58		
		Excessive alcohol consumption	0.68	0.09-5.35		
**Social cognitive variables**						
	**Physical activity**					
		Positive attitude	0.49^b^	0.25-0.96	0.50^b^	0.25-0.99
		High social support	0.80	0.30-2.13		
		High self-efficacy	0.73	0.36-1.49		
		Intention to increase physical activity	1.37	0.72-2.63		
	**Fruit and vegetable intake**					
		Positive attitude	0.63^a^	0.32-1.24		
		High social support	0.34^a^	0.08-1.46		
		High self-efficacy	1.09	0.44-2.72		
		Intention to increase intake	1.33	0.60-2.92		

	**Health indicators**					
		Overweight/obese	0.94	0.48-1.83		
		Poor/moderate general health	1.82	0.50-6.63		
		Lowest quartile mental health	0.93	0.44-1.97		
		Lowest quartile physical health	1.37	0.67-2.82		
		Elevated blood pressure	0.45^a^	0.19-1.05		
		Elevated total cholesterol level	1.06	0.55-2.03		
		Poor predicted maximum oxygen uptake	1.63	0.67-3.96		

^a^ *P* < .20, considered for inclusion in the multivariate logistic regression analysis

^b^ *P* < .05

## Discussion

In this study, we examined the use of the website component of a worksite physical activity and nutrition promotion program. In total, 43% of the participants visited the website after an email to promote website visits to view their personal health results and the personal advice based on the baseline questionnaire. Participants who did not meet the recommended level of physical activity were less likely to visit the website, whereas individuals with an elevated total cholesterol level were more likely to visit the website. Participants in the intervention group visited the website more often during a 3-month period than those in the reference group (18% versus 5%). Participants with a positive attitude toward increasing physical activity were less likely to use self-monitors for tracking their behavior and to complete the fat FFQ to receive tailored advice. Compared with male workers, more female workers visited the website to monitor their behavior and/or weight or to receive tailored advice on fat intake.

### Website Visits

Compared to previous studies, website visiting after the first email reminder was relatively high [6,8]. The face-to-face contact may have had a positive influence and may be one of the reasons for the relatively high initial number of visitors. However, website use was not optimal, since it was intended that all participants would visit the website. By not using the website component, a substantial part of the study group was not exposed to the content provided on the website. Leslie et al [31] found in a study investigating a physical activity website in the workplace setting that a comparable 46% of the participating employees visited the website at least once. There are studies, however, that have found higher levels of website usage. Ware and colleagues [13], for example, found in a study with a face-to-face contact and an Internet-delivered physical activity and weight management program that 78% of the participants were still using the website after 12 weeks. An important difference between our study and the study of Ware and colleagues is the role of the initial contact. In our study, the face-to-face contact consisted of feedback of test results and personal advice, while in the study of Ware it was a screening and an information session on how to use the Internet-delivered program. One of the explanations for the lower usage level in our study may be that people participated in the study primarily to get insight into their health status (cholesterol level and blood pressure) and that they were less interested in changing their behavior. The fact that participants could visit the website component after a series of tests and advice based on these tests in a face-to-face contact may have made it less relevant for them to visit the website to review their results and to obtain additional advice and information about a healthy lifestyle. Another explanation might be a lack of new content on the website. It has been suggested by experts as well as potential users that the provision of regular new content could be an important factor in encouraging website use [17,18].

### Correlates of Website Visits

Participants with an elevated cholesterol level were more likely to visit the website, which may indicate that visiting the website component was relevant for participants with less favorable test outcomes. In contrast, in the month after the email to promote website use was sent, individuals meeting the physical activity guideline were more likely to visit the website. Verheijden et al [8] also reported contradictory findings, with more participation among people with healthier lifestyles and among overweight or obese participants. It could be hypothesized that those with poorer outcomes on health indicators had a higher risk perception as compared with those not complying with lifestyle recommendations. However, elevated cholesterol level was the only health indicator associated with website use, and this finding was not corroborated by other health indicators such as blood pressure and self-reported health and, thus, the finding that elevated cholesterol level was associated with website use may be spurious. The finding that participants not meeting the physical activity guideline were less likely to use the website might be related to the communication to encourage the individual to change their behavior. However, this lower website use was only found in the first period and not in the period with monthly email messages. Based on our results, no consistent higher participation was found among those with healthier behaviors, and, thus, a health-based selection in website use could not be demonstrated.

### Use of Interactive Website Elements in the Intervention Condition

In line with other studies, we found that Internet access in the following 3 months was low [5-8]. Even though the 3 email reminders sent in this period resulted in a higher percentage of website visits compared with the reference group, only 18% visited the website. The difference between the reference group and the intervention group provides evidence that monthly email messages function as a prompt to visit the website; however, it may be a weak prompt. Ware et al found a high repeated participation with an Internet-delivered program using an accelerometer and weighing scale as monitoring devices [13]. The availability of such devices might increase compliance with the use of self-monitors. Experts have suggested that the possibility to monitor progress could be a factor to encourage website use [17]. In a focus group, study participants mentioned that the possibility of asking questions on a website for behavior change would increase use [32]. However, the findings of our study do not seem to support these notions. We do not know, however, why participants visited the website again in the 3-month period. Additional qualitative information of website use may shed more light on this in future studies.

Participants with a positive attitude (ie, those who thought that it would not take a lot of effort to increase physical activity and fruit and vegetables intake) were less likely to track their behavior or to obtain tailored advice on fat intake. This may indicate that they did not need the website component to visit it again. Whereas women and men did not differ with respect to website visits, more women used the website to track their behavior or to obtain tailored advice on fat intake. In a systematic review on participation in worksite health promotion programs, a higher initial participation among female workers was found except for programs offering access to a fitness centre [33]. Other studies have also reported a higher participation among women in Internet-delivered programs [2,8,34]. This may be explained by a higher interest in health issues among women [14].

### Limitations

This study has some limitations. First, 2 measures of website use are reported: website access and the use of a self-monitor and a fat questionnaire to obtain tailored advice. These measures do not provide any information as to what extent the participants actually read the available information or how much time they spent on the website. Second, because of the combination of the website component with a face-to-face contact, we cannot generalize the results to website use of programs without face-to-face contact in the worksite settings. Third, departments within workplaces instead of individuals or workplaces were randomized. Since employees do not share their work space with employees from other departments, we do not think contamination was a major issue in our study. Furthermore, the programs for the intervention and reference groups were quite similar, with both groups having the opportunity to participate in a face-to-face contact and to use the website. Therefore, it would be difficult for a participant to find out that different programs were offered. Fourth, the participation levels as well as the populations of the participating workplaces differed. Not all employees had equivalent access and use of computers and email during their workday. Therefore, we estimated for all occupations in the study population if the work is primarily done using a computer. The group spending a major part of the day with computer work was not found to have an increased website use compared with workers with less or no computer work. Strengths of the study were that the user statistics are linked to the individual level and the availability of objective health indicators.

### Conclusion

This study demonstrated that almost half of the participants used the website component of a worksite physical activity and healthy nutrition promotion program in the period after a face-to-face contact with personal advice. Monthly email messages were a prompt to visit the website. However, over the longer term, low use was found in this target group. More women than men used the website to obtain personalized advice for behavior change. No consistent higher participation was found among those with healthier behaviors. This health promotion program did not provide an indication that healthier subjects are more susceptible to health promotion.

## Abbreviations

ACSM: American College of Sports Medicine
BMI: body mass index
FFQ: food frequency questionnaire
IPAQ: International Physical Activity Questionnaire
SF-12: Short Form-12
